# Wiring the Binocular Visual Pathways

**DOI:** 10.3390/ijms20133282

**Published:** 2019-07-04

**Authors:** Verónica Murcia-Belmonte, Lynda Erskine

**Affiliations:** 1 Instituto de Neurociencias de Alicante, CSIC-UMH, 03550 San Juan de Alicante, Spain; 2 School of Medicine, Medical Sciences and Nutrition, Institute of Medical Sciences, University of Aberdeen, Foresterhill, Aberdeen, Scotland AB25 2ZD, UK

**Keywords:** retina, axon guidance, progenitor cell, neurogenesis, projection, refinement

## Abstract

Retinal ganglion cells (RGCs) extend axons out of the retina to transmit visual information to the brain. These connections are established during development through the navigation of RGC axons along a relatively long, stereotypical pathway. RGC axons exit the eye at the optic disc and extend along the optic nerves to the ventral midline of the brain, where the two nerves meet to form the optic chiasm. In animals with binocular vision, the axons face a choice at the optic chiasm—to cross the midline and project to targets on the contralateral side of the brain, or avoid crossing the midline and project to ipsilateral brain targets. Ipsilaterally and contralaterally projecting RGCs originate in disparate regions of the retina that relate to the extent of binocular overlap in the visual field. In humans virtually all RGC axons originating in temporal retina project ipsilaterally, whereas in mice, ipsilaterally projecting RGCs are confined to the peripheral ventrotemporal retina. This review will discuss recent advances in our understanding of the mechanisms regulating specification of ipsilateral versus contralateral RGCs, and the differential guidance of their axons at the optic chiasm. Recent insights into the establishment of congruent topographic maps in both brain hemispheres also will be discussed.

## 1. Introduction

**Figure 1:**
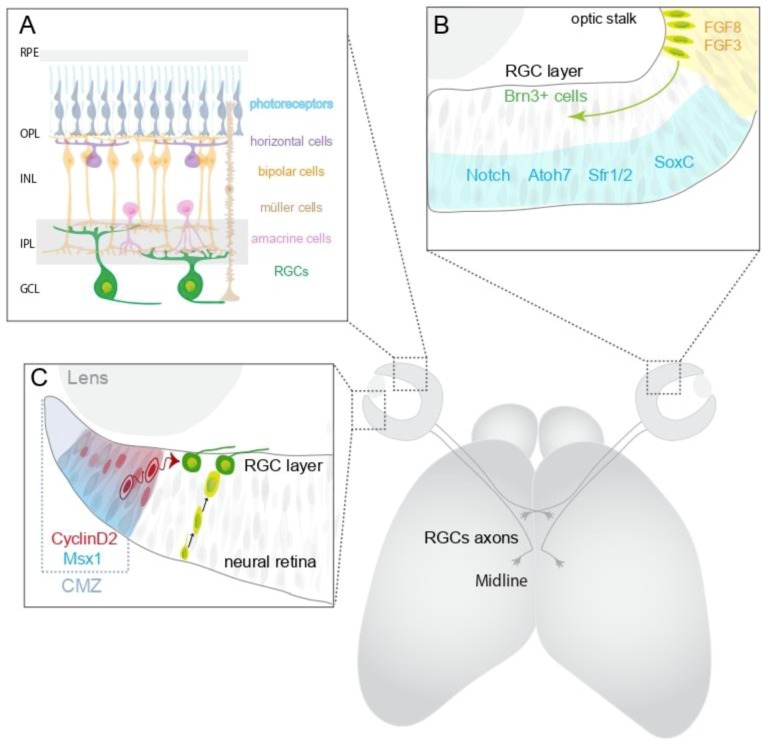
(**A**) Organization of the retina. All visual information regarding the outside world reaches the brain through the retina, which is composed of 3 layers of cells and two layers made up of connections between these cells. From inside to outside these are: the ganglion cell layer (GCL), containing nuclei of RGCs and displaced amacrine cells; the inner plexiform layer (IPL) where synapses between the bipolar cell axons and the dendrites of the ganglion and amacrine cells take place; the inner nuclear layer (INL), containing the nuclei and cell bodies of the bipolar, horizontal, and amacrine cells as well as Müller glial cells; the outer plexiform layer (OPL) where the projections of rods and cones are located and synapse with dendrites of bipolar and horizontal cells, and the outer nuclear layer (ONL), containing the cell bodies of the rods and cones which are the light detectors. The outer segments of photoreceptor cells associate with the retinal pigmented epithelium (RPE). (**B**) Transcription factors are expressed in the progenitor cells of the embryonic retina and regulate retinal neurogenesis and cell fate specification. (**C**) Regulated by Msx1 and CyclinD2, progenitor cells from the ciliary margin zone (CMZ), at the periphery of the retina, can proliferate and differentiate into all seven major retinal cell types, including ipsilaterally and contralaterally projecting RGCs.

**Figure 2:**
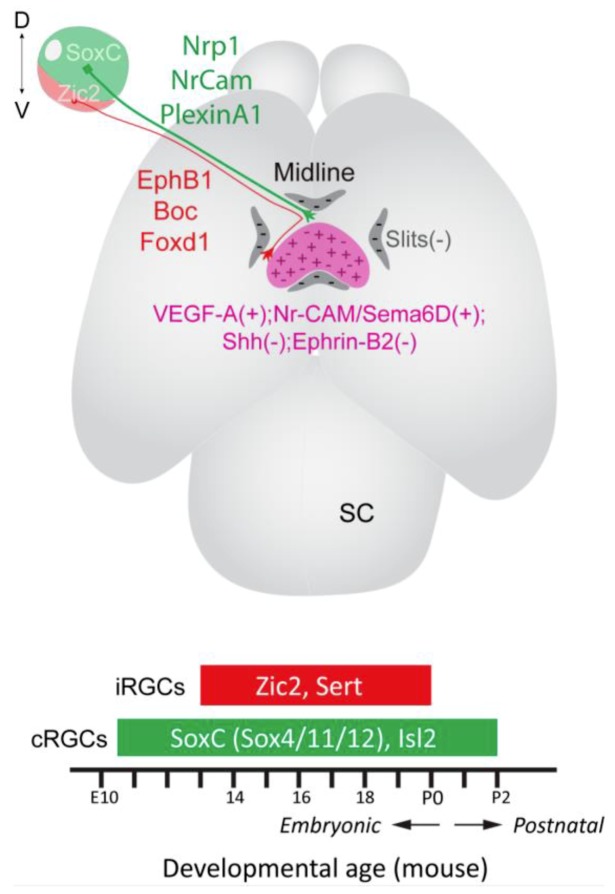
Contralaterally projecting RGCs originate throughout the retina, are specified by SoxC genes and express Nrp1, Nr-CAM and Plexin A1. Ipsilaterally projecting RGCs originate in the ventrotemporal retina in mouse, are specified by Zic2 and express EphB1, Boc and Sert. This bestows ipsilaterally and contralaterally projecting axons with differential responsiveness to cues arrayed at the chiasm midline. All RGC axons are repelled by Slits, which constrain the axons to the optic pathway. Ipsilaterally projecting RGCs are repelled away from the midline by ephrinB2, localised to radial glia at the ventral diencepahlic midline, and Shh originating from contralaterally projecting RGC axons. Crossing of contralaterally projecting axons is promoted by VEGF-A and a complex formed from Nr-CAM, Sema6D and PlexinA1. The time course during which ipsilaterally (iRGCs) and contralaterally (cRGCs) projecting RGCs are generated in mice also is shown. D, dorsal, V, ventral.

The vertebrate retina is composed of six major neuronal cells types: the photoreceptors (rods and cones), located in the outer nuclear layer, that respond to light, and the retinal interneurons (amacrine, bipolar and horizontal cells), located predominately in the inner nuclear layer that modify and relay the visual information from the photoreceptors to the retinal ganglion cells (RGCs) located mainly in the RGC layer at the inner surface of the retina (Figure 1A). RGCs, of which there are more than 30 distinct subtypes [1,2,3,4], are the only cells in the retina that extend axons out of the eye to make connections with visual targets in the brain. These connections are formed during development by the guidance of RGC axons along a relatively long, highly stereotypical pathway by cues arrayed within and around the forming optic pathway. Through differential expression of transcription factors, distinct subtypes of RGCs express diverse combination of receptors for these guidance signals enabling their accurate guidance to appropriate target regions (Figure 2).

En route to their targets, all RGC axons first have to navigate into the optic fibre layer at the inner surface of the retina where they extend in a highly directed fashion towards the optic disc, their exit point from the eye. The axons then extend in the optic nerves towards the ventral midline of the diencephalon (future hypothalamus), where the two nerves meet to form the optic chiasm. In species such as chicken and zebrafish where the two eyes have no visual overlap, all axons cross the midline at the optic chiasm to project to targets on the opposite (contralateral) side of the brain. However, in species with binocular overlap in the visual field, such as mice and ferrets, distinct subsets of axons project to targets on both the same (ipsilateral) and contralateral side of the brain [5]. From the chiasm, the axons navigate through the ipsilateral or contralateral optic tracts to their targets, of which there are over 40 in mice [6]. The primary targets are the superior colliculus (SC) and dorsal lateral geniculate nucleus (dLGN), located in the midbrain and thalamus respectively. In these targets the axons form initially a fuzzy map, in which ipsilateral and contralateral RGC axons overlap, but then segregate during the first postnatal week to innervate eye specific domains. Concurrently, in a refinement process that involves loss of RGCs and pruning of their arbors, retinal axon terminals establish connections in a topographic manner such that neighbouring RGCs connect to neighbouring target cells [7].

The proportion of RGC axons that project ipsilaterally at the optic chiasm varies widely between species and is related directly to the position of the eyes in the head, and consequently the degree of binocular overlap in the visual field [5]. In humans, with frontally located eyes, virtually all axons in the temporal half of the retina project ipsilaterally. Mice have more laterally positioned eyes, resulting in a wider field of view, but a smaller degree of binocular overlap. Accordingly, only ~3% of RGC axons, originating predominately in the far ventrotemporal (VT) crescent of the retina, project ipsilaterally in mice. Over the last few decades a number of key transcriptional and guidance programs that direct the growth of RGC axons at the mouse optic chiasm have been identified [8,9,10,11,12]. Moreover, recent gene-profiling experiments have uncovered approximately 300 genes that are differentially expressed in ipsilaterally and contralaterally projecting RGCs in mice [13], although the function of many of these genes in directing the specification and guidance of ipsilaterally and contralaterally projecting RGCs is not known currently.

In this manuscript we review our current understanding of the mechanisms controlling the specification of ipsilaterally and contralaterally projecting RGCs and the molecular mechanisms that guide the axons of these cells to their main targets in the brain. Recent work providing insights into the co-ordination of visual map formation on both sides of the brain also will be discussed.

## 2. Commitment of Retinal Progenitors to a RGC Fate

Retinal progenitors are multipotent and give rise to all of the neuronal cell types within the vertebrate retina. The different cell types are generated in overlapping waves, with RGCs the first cell type generated (Figure 1B) [14,15,16,17]. In the human retina RGCs are generated from around fetal week 5–18 [18], and in mice from embryonic day (E) 11 to postnatal day (P)0 [19,20]. A combination of intrinsic and extrinsic factors determines whether retinal progenitors remain in the cell cycle or differentiate, and the specific neuronal cell types generated. This is a highly dynamic process, with changes in the potential of individual progenitors, including restriction to particular cell fates, and alterations in the extracellular environment resulting in distinct combinations of cell types being produced at different stages of development [21,22,23,24,25,26,27,28].

A key regulator of retinal progenitor cell proliferation versus differentiation is Notch-Delta signalling (Figure 1B) [29,30]. Through lateral inhibition, Notch signalling inhibits progenitor cell differentiation and, consequently, is important for maintaining the progenitor cell pool, ensuring that sufficient numbers of progenitors are retained throughout the neurogenic period to generate all of the distinct retinal cell types [30]. Blocking Notch signalling results in large numbers of progenitors exiting the cell cycle prematurely, depleting the progenitor pool, and an overproduction of early generated retinal cell types, such as RGCs, at the expense of later generated cell types [30,31]. Through binding to and decreasing the activity of the metalloprotease ADAM10, important for propagation of Notch signalling, Secreted frizzled related protein 1 (Sfrp1) and Sfrp2 also are key regulators of retinal neurogenesis. In mice lacking *Sfrp1* and *Sfrp2* the retina is abnormally thick and contains an overabundance of early generated retinal cell types and fewer later born neurons [32]. Conversely, a positive regulator of retinal progenitor cell differentiation is the circadian clock gene *Bmal1*. In mice lacking *Bmal1* specifically in the retina, cell cycle kinetics are disrupted resulting in more retinal progenitor cells reentering the cell cycle instead of differentiating. Consequently, generation of early born retinal cell types, such as RGCs and amacrine cells, is decreased with an increase in later generated cell types [33]. Together these data demonstrate the important role that the timing of cell cycle exit plays in determining retinal progenitor cell fate, with progenitors that exit the cell cycle earlier biased towards a RGC fate. Differentiation of retinal progenitors also is regulated by a range of other extrinsic signals including factors released by differentiated cells which act back on the progenitor cells to promote or inhibit differentiation of specific cell types [34,35].

Temporal differences in retinal progenitor cell competence are driven by intrinsic changes in the progenitor cells, with progenitor cells present at early embryonic stages expressing distinct sets of transcription factors from those present during late embryonic/postnatal development [21,27]. MicroRNAs (miRNAs) are important regulators of this temporal change in retinal progenitor cell competence. Conditional deletion from the retina of Dicer, essential for processing of mature miRNAs, impairs the early-to-late switch in retinal progenitor cell competence. In mouse conditional *dicer* mutants markers of late generated progenitors are not expressed, late generated cells types are not produced and generation of early cell types, such as RGCs, is increased due to a prolonged period of genesis. Key miRNAs important for the early-to-late switch in retinal progenitor cell competence are let-7, microRNA-125 and micro-RNA-9. Ectopic early expression of these microRNAs accelerates the generation of late-born retinal cell types, whereas blocking function of these miRNAs inhibits production late generated cell types [26,27,28,36].

Retinal neurogenesis is triggered initially in central retina by the combined activity of FGF8 and FGF3 emanating from the optic stalk (Figure 1B) [37,38]. Subsequently, differentiation propagates across the entire retina in central-to-peripheral waves (Figure 1B) [39]. Commitment of retinal progenitors to a RGC fate occurs during or just after the final cell division [18,21,40,41]. The transcription factor *Atoh7* (*Math5*) is necessary but not sufficient for commitment to a RGC fate. *Atoh7*-expressing cells give rise to multiple retinal cell types, and only a small percentage of *Atoh7*-positive progenitors (~11%) differentiate into RGCs. Moreover, RGC can be generated by *Atoh7*-negative progenitors, particularly at early stages of development [21,42]. However, mice lacking *Atoh7* (*Math5*) lack over 80% of RGCs, with cells instead adopting an amacrine or cone photoreceptor fate [43,44]. This has led to the idea that *Atoh7* is required both cell autonomously and in neighbouring cells to establish RGC competence, with other intrinsic and extrinsic factors required for commitment to the RGC fate. Nearly 1500 transcripts that are expressed differentially between *Atoh7*-positive and -negative progenitors have been identified, and encode for factors involved in a diverse range of processes including neuronal differentiation and development, cell migration, neuron projection, and neurotransmission [45]. Key downstream targets of *Atoh7* include the transcription factors Brn3b (Pouf42), and Islet-1 (Isl1) which act in parallel pathways to regulate morphological differentiation and survival of RGCs (Figure 1B) [42,44,46,47]. In combination Brn3b and Isl1 appear sufficient to specify RGC fate. Ectopic expression of *Brn3b* and *Isl1* in *Atoh7* null retinas drives essentially normal differentiation of RGCs [48]. The SoxC family members Sox4 and Sox11 also are both necessary and sufficient for generation of RGCs. In mice lacking *Sox4* and *Sox11* specifically in the retina, generation of RGCs is decreased significantly. Conversely, ectopically expressing *Sox4* and *Sox11* in E14.5 retinal cells and human iPSCs in culture is sufficient to promote RGC differentiation [49].

## 3. Production of Retinal Cells by the Mammalian Ciliary Margin Zone

The ciliary margin zone in fish and amphibians is a source of retinal stem cells, producing new retinal cells to support growth of the retina throughout life [50,51,52]. However, until recently it was thought that this ability of the ciliary margin zone to produce retinal neurons was lost in mammals. Taking advantage of the fact that *Msx1* is expressed specifically by ciliary margin zone progenitor cells, lineage tracing using a tamoxifen-inducible *Msx1Cre^ERT2^; RYFP* reporter mouse identified an *Msx1*-positive progenitor cell population in the embryonic mouse ciliary margin zone that gives rise to both retinal neurons and the non-neural ciliary epithelium of the iris and ciliary body [53]. Independently, live imaging of eGFP-labelled ciliary margin zone cells in the embryonic mouse retina identified cells born from ciliary margin progenitors that transit to the RGC layer of the neural retina [54] (Figure 1C). These ciliary margin progenitors are capable of generating all retinal neuronal cell types, with the switch between neural and non-neuronal fates regulated by the asymmetric inheritance of numb in daughter cells. The ability of the ciliary margin progenitors to generate retinal neurons appears to be lost at postnatal stages [53]. However, whether injury or disease can re-stimulate neurogenesis by these cells has not been established.

## 4. Specification of RGC Subtype Identity

Based on morphology, functional properties, presynaptic partners and central projection patterns, approximately 30 different RGC subtypes have been characterised in the mouse retina [1,2,3]. Recent single cell RNA-seq analyses of RGCs have placed the potential number of distinct RGC subtypes at around 40 [4]. Differentiation of nascent RGCs into identifiable RGC subtypes occurs postnatally and is regulated by differential transcription factor expression. For example, the T-box transcription factor T-brain 1 (Tbr1) is expressed perinatally in a subset of RGCs and is required for the formation and maintenance of two distinct RGC subtypes—orientation-selective J-RGCs and a group of OFF-sustained RGCs [55]. The transcription factors Brn3a, Brn3b, and Brn3c form a combinatorial code important for development of specific RGC subtypes [56,57,58,59]. Melanopsin expression in intrinsically photosensitive RGCs (IpRGCs) is regulated by Isl1 [59]. Downstream targets of the Brn3 transcription factors have been identified in RGCs and include a range of other transcription factors and cell surface molecules that are expressed differentially by distinct RGC subtypes [58,60]. These experiments have provided important insights into the mechanisms controlling RGC subtype identity. However, our understanding is still relatively rudimentary, and much still remains to be unravelled.

Based on single cell RNA-seq transcriptome profiling, most subtypes of RGCs appear to be present proportionally in both eyes, although a few RGC subtypes predominate in one eye compared to the other [4]. Axonal tracing experiments also have demonstrated that ipsilaterally projecting RGCs are composed of more than one subclass of RGCs [61]. This suggests that the mechanisms controlling RGC laterality are distinct from those regulating RGC subtype identity and occur similarly in the majority of nascent RGC subtypes.

## 5. Specification of Ipsilaterally versus Contralaterally Projecting RGCs

### 5.1. Temporal and Spatial Dynamics of Generation of Ipsilaterally and Contralaterally Projecting RGCs

Generation of ipsilaterally versus contralaterally projecting RGC is a highly dynamic process and differs both temporally and spatially in the mouse retina (Figure 2). The first RGCs in mouse are generated at ~E11 in dorsocentral retina and this initial central patch gives rise to both contralaterally projecting RGCs and a transient, early born population of ipsilaterally projecting RGCs [19,62,63]. The axons of these early born ipsilaterally projecting RGCs do not approach the midline, but extend straight into the ipsilateral optic tract [64], appear to employ distinct guidance mechanisms to the later generated, permanent ipsilaterally projecting axons [9,12], and are mainly eliminated before reaching brain target regions [65]. From E11.5 until birth generation of contralaterally projecting RGCs spreads peripherally throughout the retina. Until recently it was thought that production of contralaterally projecting RGCs was essentially excluded until ~E15.5 from the far ventrotemporal region of the mouse retina (ventrotemporal crescent), the site of origin of the permanent ipsilateral projection [19]. It also was thought that the production of the permanent ipsilaterally projecting RGCs in the ventrotemporal crescent did not occur until ~E14.5 and ceased by ~E17.5, with the ventrotemporal crescent now giving rise almost exclusively to a late-generated population of Islet 2 (Isl2)-dependent contralaterally projecting RGCs [11,19,66]. However, recent birth dating experiments in combination with cell type-specific markers for ipsilaterally versus contralaterally projecting RGCs have challenged these ideas [67]. These experiments demonstrated that neurogenesis in ventrotemporal retina is delayed compared to dorsotemporal retina. However, production of ipsilaterally projecting RGCs in ventrotemporal retina begins earlier than thought and peaks at E13.5. Moreover, production of contralaterally projecting RGCs in ventrotemporal retina occurs in an overlapping wave with the generation of ipsilaterally projecting RGCs, beginning around E14.5. The so called “late-generated” contralaterally projecting RGCs also originate in ventrotemporal retina earlier than previously thought, and are born at around E15.5 [67]. There appears though to be a lag between generation of these late born contralaterally projecting RGCs and expression of Isl2 which is not upregulated in ventrotemporal retina until E17.5 [66]. Analysis of cell cycle kinetics also demonstrated that cell cycle exit is slower in ventrotemporal retina than other retinal regions [67]. Taken as a whole, these findings suggest that, similar to specification of the distinct retinal cell types, cell cycle dynamics and timing of birth of RGCs within ventrotemporal retina is important for the generation of ipsilaterally versus contralaterally projecting RGCs. In support of this idea, delayed neurogenesis has been linked to the ~50% reduction in generation of ipsilaterally projecting RGCs in albino retinas [68,69,70,71].

Within the ventrotemporal crescent, both ipsilaterally and contralaterally projecting RGCs have a dual origin from neural retina and ciliary margin zone progenitors [54]. The cell cycle regulator CyclinD2 is a key regulator of RGC genesis from ciliary margin progenitors. At early embryonic stages, CyclinD2 is expressed at high levels in the ciliary margin of ventral retina relative to other retinal regions (Figure 1C). In E13.5 and E14.5 mice lacking CyclinD2, the number of mitotic cells is not altered in the neural retina but decreased significantly in the ventral ciliary margin zone. Associated with this is a decreased production of both ipsilaterally and contralaterally projecting RGCs in peripheral ventral retina (Figure 1C). Moreover, in albino mice, which have fewer ipsilaterally projecting RGCs, the number of CyclinD2-positive cells in the ventral ciliary margin zone is decreased compared to pigmented mice [54]. These data point to an important role for ciliary margin zone progenitors and CyclinD2 in production of both ipsilaterally and contralaterally projecting RGCs in ventrotemporal retina (Figure 1C). However, much still remains to be established. For example, the mechanisms that co-ordinate RGC genesis from ciliary margin and neural retina progenitors to ensure the correct proportion of ipsilaterally versus contralaterally projecting RGCs in ventrotemporal retina are not known. Moreover, it is not known if individual progenitors in either the ciliary margin zone or neural retina are restricted to produce only ipsilaterally or contralaterally projecting RGCs, or can produce both subtypes either from the same cell division or at distinct times in development.

### 5.2. Specification of Ipsilaterally Projecting RGCs

The transcription factor Zic2 is expressed specifically by ipsilaterally projecting RGCs and is necessary for the establishment of ipsilateral identity (Figure 2). In mice, expression of Zic2 is first detected in RGCs in ventrotemporal retina at E14.5, and begins to decline from E17.5 when the generation of ipsilaterally projecting RGCs terminates. Albino mice have fewer Zic2-positive RGCs than pigmented mice, correlating with the reduced size of the ipsilateral projection in albinos. Conversely, in ferrets, which have a larger binocular overlap in the visual field, more Zic2-positive RGCs are found compared to mice, whereas no Zic2-positive RGCs are present in species such as chicken and *Xenopus* tadpoles with purely crossed chiasmatic projections. In hypomorphic *Zic2* mouse mutants, with reduced levels of *Zic2*, substantially fewer ipsilaterally projecting RGCs are generated [9] demonstrating that Zic2 is necessary for specification of ipsilaterally projecting RGCs.

Zic2 also is sufficient to drive ipsilateral identity. Ectopic expression of *Zic2* in central retina, which normally gives rise to contralaterally projecting RGCs, results in a proportion of central RGC axons now projecting ipsilaterally at the optic chiasm [72]. Zic2 controls ipsilateral identity, at least in part, by regulating expression of EphB1, important for repulsion of ipsilaterally projecting axons away from the chiasm midline ([12]; see below). Expression of *Zic2* in ipsilaterally projecting RGCs precedes expression of *EphB1*, and ectopic expression of *Zic2* is sufficient to drive *EphB1* expression in central RGCs. Moreover, ectopic *Zic2* expression in central retina of *Ephb1*-deficient mice results in many fewer Zic2-positive central RGCs projecting ipsilaterally than in wild-type mice [72].

The mechanisms that restrict *Zic2*-expression to a subset of ventrotemporal RGCs are beginning to be unravelled. Expression of the transcription factor *Foxd1* precedes *Zic2*-expression in ventrotemporal retina, and in mice lacking *Foxd1 Zic2* expression is lost in ventrotemporal retina [73,74]. Ectopically expressing *Foxd1* in central retina results in axons misrouting into the ipsilateral optic tract [73]. Together these findings indicate that Foxd1 may act upstream of Zic2 to specify ipsilateral identity. However, in mice lacking *Foxd1* a larger than normal ipsilateral projection develops, rather than a loss of ipsilateral projections as expected if Foxd1 is essential for specifying ipsilateral identity. This increase in ipsilateral projections in *Foxd1* mutants may reflect a parallel role for Foxd1 in patterning the developing chiasmatic region, and result from axons of contralaterally-specific RGCs failing to cross the midline rather than defective specification of ipsilaterally versus contralaterally projecting RGCs [74].

Recently, Wnt signalling in the peripheral RPE has been implicated as a negative regulator of ipsilateral identity. *Wnt2b* is expressed in the peripheral retina and RPE and is important for development of non-neural peripheral retinal structures, such as the ciliary body and iris [75]. At E15.5, the peak period of RGC axon divergence at the optic chiasm, expression of *Wnt2b* in the peripheral RPE is expanded centrally in albino retinas compared to pigmented mice. Feeding pregnant mice lithium chloride, an activator of Wnt signalling, decreased the number of Zic2-positive RGCs in E15.5 pigmented retinas by ~50%, to levels similar to those found normally in albino retinas. This reduction in production of ipsilaterally projecting RGCs occurred in the absence of obvious changes in peripheral retina patterning and retinal progenitor cell proliferation [76]. However, whether cell cycle dynamics is altered in retinal progenitor cells by lithium treatment, or if Zic2 is a direct target of Wnt signalling in the retina has not been established. Further work therefore will be required to establish the mechanisms by which activation of Wnt signalling regulates production of ipsilaterally projecting RGCs.

A downstream effector of Zic2 important for maintenance of ipsilateral identity in RGCs is Boc (brother of Cdon), a receptor for Sonic hedgehog (Shh). *Boc* is expressed specifically by ipsilaterally projecting RGCs, whereas Shh is produced by contralaterally projecting RGCs. In hypomorphic *Zic2* mutants and mice lacking *Foxd1,* expression of *Boc* in ipsilaterally projecting RGCs is decreased substantially [77]. Loss of Boc results in decreased ipsilateral projections at the optic chiasm [77,78]. However, ectopic expression of *Boc* is not sufficient to drive acquisition of ipsilateral identity. Instead Boc appears to act in ventrotemporal retina in a feedback loop with Zic2 to increase sensitivity of ipsilaterally projecting RGCs to Shh, which is produced by neighbouring contralaterally projecting RGCs. In turn, Zic2 represses *Shh* expression. In combination this is important for limiting production of contralaterally projecting RGCs and maintaining the correct number of ipsilaterally projecting RGC. Disruption of this loop results in more contralaterally projecting RGCs being produced in ventrotemporal retina, at the expense of ipsilaterally projecting RGCs [77].

Another potential downstream effector of Zic2 is Ten-M2, a member of the highly conserved Ten-m/Odz/Teneurin family of transmembrane glycoproteins. In mice Ten-M2 is expressed throughout the RGC layer, and in mice lacking *Ten-M2* fewer RGC axons than normal project ipsilaterally. The number of Zic-2-positive RGCs is not altered obviously in the absence of Ten-M2, but expression of *EphB1* is decreased substantially within peripheral ventral retina. These finding suggest that Ten-M2 controls axon laterality at the chiasm by regulating *EphB1* expression in peripheral ventral retina [79]. However, further work will be required to establish if Ten-M2 acts in the same or a parallel pathway to Zic2 to regulate *EphB1* expression.

### 5.3. Specification of Contralaterally Projecting RGCs

The first factor identified as being important for the production of contralaterally projecting RGCs was the transcription factor Isl2. In mice, Isl2 is expressed by ~40% of RGCs that in the adult retina constitute a distinct subpopulation of RGCs [66,80]. Although expressed specifically in contralaterally projecting RGCs distributed throughout the developing retina, loss of *Isl2* results in an increase in ipsilateral projections that originate specifically in ventrotemporal retina. Associated with this, the number of Zic2-positive RGCs in ventrotemporal retina is increased in *Isl2* mutants. Based on these findings it has been proposed that Isl2 acts to repress ipsilateral identity in Isl2-expressing RGCs located in ventrotemporal retina rather than actively specifying contralateral identity [66]. More recently, the SoxC family genes, *Sox4*, *Sox11* and *Sox12,* have been identified as key regulators of contralateral RGC differentiation and specification (Figure 2). Knocking down SoxC expression in E14.5 retinas inhibits differentiation of contralaterally projecting RGCs in vitro and in vivo [81], reflecting the role of these factors in driving generation of RGCs [49]. Deletion at later stages (E16.5) enables contralaterally projecting RGCs to form, but impairs guidance of their axons at the optic chiasm, with many projecting ectopically into the ipsilateral optic tract. Associated with these guidance defects, expression of PlexinA1 and Nr-CAM (neuronal cell adhesion molecule), important for contralateral growth at the optic chiasm ([10]; see below), is decreased in contralaterally projecting RGCs following knockdown of *SoxC* genes [81]. The transcription factor Brn3a also localises specifically to contralaterally projecting RGCs [82]. However, whether Brn3a is necessary for the specification of contralaterally projecting RGCs remains to be established.

## 6. Mechanisms Controlling Differential Guidance of RGC Axons to Targets on Both Sides of the Brain

Inhibitory cues, such as Slits, surround the developing optic pathway and help channel both ipsilaterally and contralaterally projecting RGC axons towards their correct decussation point on the ventral diencephalic midline [83,84]. Driven by their distinct transcription factor expression, ipsilaterally and contralaterally projecting RGCs express diverse combinations of guidance cue receptors, enabling different responses to cues arrayed at the ventral diencephalon (chiasmatic) midline, and divergence to targets on both sides of the brain (Figure 2).

### 6.1. Guidance Molecules Directing RGC Axons Ipsilaterally at the Optic Chiasm

First demonstrated in *Xenopus*, a key regulator of ipsilateral growth at the optic chiasm is ephrin-B signalling. In *Xenopus* tadpoles, with laterally positioned eyes and a completely crossed chiasmatic projection, ephrin-Bs are not detected at the chiasmatic midline. However, at metamorphosis, when the eyes move centrally and binocularity develops, ephrin-B begins to be localised at the diencephalic midline. Ephrin-B expression at the chiasm midline also correlates with the presence of ipsilateral projections in other species—no ephrin-B expression is detected at the chiasm midline of chicken and zebrafish, which lack ipsilateral RGC projections, but is present at the ventral diencephalon in mouse. Moreover, ectopic expression of ephrin-B in *Xenopus* tadpoles is sufficient to drive generation of a precocious ipsilateral projection [85]. Subsequent studies in mice identified the precise family members involved and demonstrated that ephrin-B signalling is necessary for ipsilateral growth of RGC axons at the optic chiasm. In mice, *EphB1* is expressed by ipsilaterally projecting RGCs and its ligand *ephrin-B2* is expressed specifically at the ventral midline site where the optic chiasm develops. Moreover, expression of *ephrin-B2* at the chiasm midline is restricted temporally to the period when the ipsilateral projection is actively generated. Through inhibitory signalling that deflects ipsilaterally projecting axons away from the midline, EphB1 and ephrin-B2 are essential for driving axon growth ipsilaterally. In mice lacking *EphB1* or in a cultured semi-intact preparation of the retina, optic nerves and ventral diencephalon in which ephrin-B2 signalling is blocked, the number of axons projecting ipsilaterally at the optic chiasm is decreased substantially [12]. However, some axons still project ipsilaterally following loss of EphB1 or ephrin-B2 signalling, indicating that other factors also are involved in directing ipsilateral growth.

In addition to controlling the balance in production of ipsilaterally and contralaterally projecting RGCs [77], Shh helps directs ipsilateral axon growth at the optic chiasm. Interestingly, the source of Shh important for ipsilateral growth at the chiasm is contralaterally projecting axons rather than cells of the ventral diencephalon midline. *Shh* is not expressed at the chiasm midline. However, Shh protein is produced by contralaterally projecting RGCs and transported along their axons where it accumulates at the optic chiasm before ipsilaterally projecting RGC axons arrive in this region. *In vitro*, ipsilaterally projecting axons are repelled by contralaterally projecting axons and this inhibitory signalling is mediated by the Shh receptor Boc. Knocking down *Shh* in one eye decreases ipsilateral projections originating from the other eye, demonstrating that Shh acts outwith the eye to regulate guidance at the optic chiasm [86]. It has long been known that interactions between axons from both eyes are important for correct decussation at the optic chiasm [87,88,89]. These findings provide a molecular basis for the role of axons from the opposite eye in regulating development of ipsilateral projections: Shh produced by contralaterally projecting RGCs acts on Boc-positive ipsilaterally projecting axons to help drive axon growth into the ipsilateral optic tract [86].

### 6.2. Guidance Molecules Promoting Midline Crossing at the Optic Chiasm

A key regulator of midline crossing at the mouse optic chiasm is the cell adhesion molecule Nr-CAM. In mice, Nr-CAM is expressed both by contralaterally projecting RGCs and by radial glial cells at the ventral diencephalon midline [11,90]. Loss of Nr-CAM alone results in defective midline crossing of the late-generated contralaterally projecting RGCs located in ventrotemporal retina [11]. However, in combination with Sema6D and PlexinA1, Nr-CAM also helps promote crossing of earlier generated RGC axons. Nr-CAM and Sema6D both are expressed by midline glial cells of the ventral diencephalon, whereas PlexinA1 is expressed by an early born population of diencephalic neurons located directly posterior to the developing optic chiasm. Nr-CAM and PlexinA1 also are expressed by contralaterally projecting RGCs and mediate axonal responses to Sema6D. In vitro, when presented alone, Sema6D inhibits outgrowth of contralaterally projecting RGCs. However, when Sema6D is presented together with Nr-CAM and PlexinA1, as occurs in vivo, this repulsive effect on contralateral RGC axons is converted to growth promotion. Loss of Nr-CAM or Plexin-A1 individually has little impact on axon projections at the optic chiasm. In contrast, loss of Sema6D or both Nr-CAM and PlexinA1 results in axon defasciculation and an increased number of RGC axons projecting ipsilaterally at the optic chiasm. These increased ipsilateral projections originate from regions of the retina that give rise normally to contralaterally projecting RGCs, suggesting that in the absence of Sema6D or PlexinA1/Nr-CAM, axons of some contralaterally specified RGCs fail to cross the midline and instead project aberrantly into the ipsilateral optic tract [10].

Independent of their role in blood vessels, Neuropilin-1 (NRP1)-binding isoforms of vascular endothelial growth factor (VEGF-A) also help promote contralateral growth at the mouse optic chiasm. Through alternative splicing, the *Vegfa* gene gives rise to a range of different isoforms that differ in their heparin-binding affinity and ability to bind and signal through NRP1 [91,92]. VEGF_164_ and VEGF_188_ bind NRP1 in vivo and can evoke NRP-1 dependent signalling in neurons, whereas the shorter VEGF_120_ isoform cannot mediate NRP1-dependent signalling [92]. During the period when the optic chiasm is actively developing, all 3 VEGF-A isoforms are expressed at the ventral diencephalon midline, whereas NRP1 is expressed by contralaterally projecting RGCs, in addition to endothelial cells [8,92]. VEGF-A provides growth promoting and chemoattractive signals to NRP-positive contralaterally projecting RGCs in vitro and both VEGF_164_ and VEGF_188_ are sufficient for contralateral growth of RGC axons at the optic chiasm [8,92]. In mice lacking NRP1 or expressing only the non-NRP1-binding VEGF_120_ isoform presumptive contralaterally projecting axons fail to cross the midline and instead enter the ipsilateral optic tract. Fasciculation of both the ipsilateral and contralateral optic tracts also is perturbed [8]. Conditional knockout of *Nrp1* from RGCs confirmed that NRP1 is required autonomously in RGCs for contralateral growth at the optic chiasm [93]. In contrast, loss of *Nrp1* specifically from endothelial cells does not impair midline crossing at the optic chiasm but impacts indirectly on RGC axon organisation due to aberrant blood vessel development. In mice lacking *Nrp1* in endothelial cells aberrant vessels develop at the ventral diencephalon midline and within the optic tracts. These vessels form a physical barrier to RGC axon progression. However, RGC axons are able to activate mechanisms that enable them to navigate around the aberrant vessels and progress towards their targets. The deviation of the axons from their normal straight path as they navigate around the vessels results in the appearance of gaps or holes in the axons bundles, but otherwise relatively normal development of the optic chiasm and optic tracts [93]. Further work will be required to establish the mechanisms that normally exclude vessels from the diencephalon midline despite the strong expression of VEGF-A, a potent promotor of angiogenesis, in this region.

Interestingly, although NRP1 is not expressed by ipsilaterally projecting RGCs [8], both the ipsilateral and contralateral optic tracts are defasciculated in RGC-specific *Nrp1* mutants [93]. The lack of NRP1 expression in ipsilaterally projecting RGCs makes it unlikely that NRP1 controls axon fasciculation directly. Instead, the axon defasciculation in the absence of NRP1 may reflect the misrouting of contralaterally-specified RGC axons into the ipsilateral optic tract. In vitro ipsilaterally projecting RGC axons self-fasciculate to a greater extent than contralaterally projecting axons [94], and in vivo, ipsilaterally and contralaterally-specified RGC axons from opposite eyes are spatially segregated within the same optic tract [65,95]. Moreover, this segregation of ipsilaterally and contralaterally-specified RGC axons is maintained in *Ephb1* mutants in which many ipsilaterally-specified RGC axons are misrouted into the contralateral optic tract [94]. Accordingly, misrouting of contralaterally-specified RGC axons into the ipsilateral optic tract may disrupt tract organisation due to segregation of these misrouted axons from the ipsilaterally-specified RGC axons. In support of this idea, the ipsilateral optic tract of all known mouse mutants with excessive ipsilateral projections appear defasciculated [8,10,96].

NRP1 also is required for contralateral growth of RGC axons in zebrafish which normally have a purely crossed chiasmatic projection. Interestingly, in this species the identified NRP1 ligand is a member of the class 3 semaphorin family. *Nrp1a* is expressed by zebrafish RGC axons as they extend towards and through the chiasm midline, whereas Sema3D is expressed in the ventral diencephalon. Morpholino knockdown of *Nrp1a* or *Sema3d* results in some axons aberrantly projecting ipsilaterally [97,98]. Overexpression of *Sema3d* also results in aberrant ipsilateral projections, suggesting that the precise level of Sema3D is important for mediating midline crossing [98]. NRP1 therefore has a conserved role in mediating contralateral growth at the optic chiasm of both mice and zebrafish. However, the NRP1 ligand driving contralateral growth appears to be different: Sema3D in zebrafish and VEGF-A in mice. Mice lacking semaphorin-signalling through NRPs do not display defects in midline guidance at the optic chiasm, indicating that Sema3D is not essential for contralateral growth at the mouse optic chiasm [8]. However, whether VEGF-A is required for RGC axon guidance at the zebrafish optic chiasm has not been established. The LRR receptor Islr2 also appears to mediate different roles at the zebrafish and mouse optic chiasm. Zebrafish lacking *Islr2*, which is expressed normally by RGCs, develop ectopic ipsilateral projections. However, mice lacking *Islr2* do not display obvious defects in axon crossing at the optic chiasm, but exhibit defects in axon fasciculation and an increase in RGC axons projecting into the contralateral optic nerve [99].

miRNAs also are important for regulating contralateral growth at the mouse optic chiasm. In conditional mouse mutants lacking *Dicer* in the retina and ventral forebrain, the proportion of axons projecting ipsilaterally is increased substantially. RGC axons also make other guidance errors in conditional *Dicer* mutants, including increased projections into the contralateral optic nerve, and wandering away from the optic pathway into aberrant regions of the brain. Zic2 localisation is not altered obviously in the retina of conditional *Dicer* mutants, indicating that miRNAs are likely involved in regulating the guidance of RGC axons rather than specification of ipsilaterally or contralaterally projecting RGCs [96]. However, the miRNA target(s) important for regulating guidance at the optic chiasm remain to be established.

## 7. The Establishment of Congruent Topographic Maps in Both Brain Hemispheres

### 7.1. RGCs Projections in the Superior Colliculus

**Figure 3:**
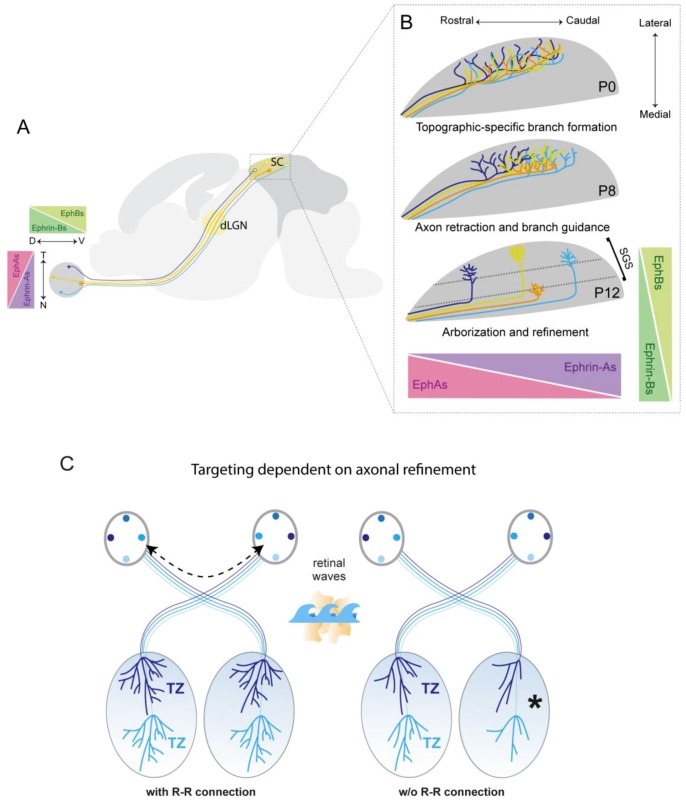
Establishment of RGC axon organisation in the mouse superior colliculus. (**A**) RGC axons map topographically in the superficial superior colliculus. (**B**) Retinotopic map formation is regulated by the interaction of ephrin/Eph gradients. EphAs are expressed in an increasing nasal-temporal gradient in the retina, whereas ephrinA ligands are expressed in an increasing anterior-posterior gradient in the superior colliculus. In the dorsal-ventral axis, a gradient of EphBs is expressed highest in the ventral retina, while a gradient of ephrinBs is highest in the medial superior colliculus. RGC axons initially overshoot their termination zone then retract by the elimination of overextended branches. This refinement process is regulated by activity as well as molecular factors. (**C**) Based on a mathematical model, a retina–retina (R–R) connection may enable the synchronization of retinal waves from each eye and the establishment of bilaterally congruent maps through symmetrical refinement. In the absence of a retina-retina connection (w/o R–R connection), retinal refinement in each hemisphere may be different generating a non-continuous topographic map.

The main RGC recipient nuclei in the brain are the dorsal lateral geniculate nucleus and the superior colliculus (Figure 3A). The superior colliculus plays an important role in visual information processing in the mouse visual system including coordinating eye and head movements [100], suspension of locomotion [101], and escape or freezing in response to a looming object [102,103]. In the mouse visual system topographic mapping is established progressively during the first two postnatal weeks. No obvious order exists for nasal-temporal axons on reaching the colliculus. However, sorting of dorsal-ventral axons is established already in the optic tracts [104]. In zebrafish, the cytoplasmic FRM1-interacting proteins CYF1P1 and CYF1P2 are important for this sorting [105]. Initially, RGC axons arborise throughout the superior colliculus. Then, after a process of removing inappropriate axonal branches in a topographic manner, RGC axons’ termination zones mature and form a point-to-point representation of the retina [106,107,108].

The superior colliculus receives projections from 85% to 90% of RGCs in mice [109] and is organized into several synaptic layers, each of which has distinct sources of innervation [110,111]. The most superficial lamina of the superior colliculus, the stratum griseum superficiale (SGS), receives direct RGC inputs from the contralateral retina in its most superficial region. Inputs from the ipsilateral retina arrive to the lower SGS lamina. [112,113]. Ipsilateral and contralateral RGCs also project to different areas in the superior colliculus depending on their topographic location in the retina. While ipsilaterally projecting RGC axons are located at the ventrotemporal periphery of the mouse retina, their axons are topographically located in the rostromedial superior colliculus. In contrast, contralaterally projecting RGC axons populate the whole retina and their axons fill the superior colliculus in a complementary pattern with the ipsilateral RGC axons.

The most studied mechanisms by which RGC axons sort to form a topographic map in the superior colliculus is the combination of Eph/ephrin signaling and patterned spontaneous retinal activity [7,114]. For example, EphA receptor tyrosine kinases and their ligands, ephrin-As, are expressed in gradients along the nasal-temporal axis of the retina and throughout the anterior-posterior axis of the superior colliculus [115,116,117]. EphA/ephrin-A signaling is essential for normal map development, positioning the temporal RGC axons in the anterior superior colliculus, and nasal RGC axons in the posterior superior colliculus [116,118,119,120]. Disruption of EphA/ephrin-A signaling results in RGC axons having ectopic termination zones and superior colliculus neurons having topographically incorrect receptive field locations along the anterior-posterior axis of the superior colliculus [121,122,123,124,125,126] (Figure 3B). Mapping along the medial-lateral axis of the superior colliculus also is regulated by Eph-ephrin signalling, but in this case is mediated by members of the EphB/ephrinB families. In mice, *EphB1* and *EphB2* are expressed in a high ventral to low dorsal gradient in the retina and *ephrinB1* in a high medial to low lateral gradient in the superior colliculus. In mice lacking EphBs or ephrinBs ectopic termination zones are formed, lateral to the appropriate position [127,128]. Depending on their concentration level, ephrinBs may mediate both attractive and repellent responses of RGC axons important of mapping along the medial-lateral axis of the colliculus [129]. Moreover, both forward and reverse EphB/ephrinB signalling have been implicated in regulating medial-lateral mapping [128].

### 7.2. Refinement of RGC Axon Terminals

RGC axon terminals undergo an extensive refinement at postnatal stages in a process depending on spontaneous activity generated in the retina before eye opening to form appropriate topographic and eye-specific maps [130]. Ipsilateral and contralateral projections overlap at birth and then segregate to form eye-specific domains during the first postnatal week [131,132]. Achieving precise retinotopy involves the retraction of retinal projections that have initially overshot their termination zone, branching at the proper location, and axonal arbor refinement to focus the termination zone [125,129,133]. Synchronized neuronal activity is required for retinocollicular map formation [129]. During development the retina generates waves of spontaneous activity that propagate across the retina [134,135]. Although initially thought to initiate randomly throughout the retina, recent data points to a preferential origin of waves in the ventrotemporal region of the mouse retina, the sight of binocular overlap in the visual field [134]. Perturbing spontaneous retinal activity leads to defects in both eye-specific segregation and retinotopic refinement [125,129,136,137,138,139]. For instance, the ectopic expression of the potassium channel Kir2.1 in individual embryonic neurons to block spontaneous activity *in vivo* does not affect neuronal identity specification or axon pathfinding during the development of topographic maps, but impairs axon branching and pruning once axonal RGCs growth cones reach their correct topographic position in the target tissues [136]. In addition to being important for specification of ipsilaterally projecting RGCs, Zic2 also helps control the eye specific refinement of RGC axons in visual targets through regulating directly the expression of the serotonin transporter (Sert) important for activity-dependent refinement in visual targets [140].

## 8. Guidance Mechanisms at the Optic Chiasm Important for Symmetry of Topographic Maps on Both Sides of the Brain

Spontaneous activity originating in the retina is propagated throughout the visual system and results in corresponding waves of activity in the superior colliculus. Although thought to originate autonomously in each eye, a subset of waves propagates in a highly temporally and spatially correlated manner in both hemispheres of the superior colliculus. One potential function proposed for this synchronisation of activity is to ensure bilateral congruent refinement of RGC arbors within the colliculus [134]. A direct connection between both retinas could enable synchronisation of retinal waves to occur. In support of the idea that activity in one eye impacts on wave generation in the other eye, removing one eye from ferrets at birth alters wave dynamics in the remaining eye [141]. However, although small numbers of retina-retina axons have been detected in the developing visual system of a range of species [142,143,144,145,146], until very recently they were considered likely artifacts of the labelling method or the result of projection errors during development. Using eGFP to label small populations of RGCs in the mouse retina, the existence of RGC axons that project normally into the contralateral optic nerve and reach the contralateral retina was demonstrated unequivocally during the perinatal period. These axons originate in the ventronasal retina and, in the opposite eye, their axons come in close contact to starburst amacrine cells [147]. However, further work is required to further explore the interaction of retina-retina axons and amacrine cells.

Active guidance mediated by Netrin-1 expressed ventrally at the chiasm and Unc5c expressed in ventronasal RGCs prevents a subset of RGC axons from entering the optic tracts, and instead drives them into the contralateral optic nerve. In mice lacking *Unc5c* or following specific knockdown of *Unc5c* in the retina, the number of axons projecting into the contralateral optic nerve is decreased substantially. Conversely, ectopic expression of Unc5c in RGCs is sufficient to drive axon growth towards the opposite eye. *Unc5c* is expressed specifically by contralaterally projecting RGCs and expression of *Unc5c* appears to be negatively regulated by Zic2. In *Zic2* hypomorphic mutants the *Unc5c* expression domain is expanded, whereas overexpressing Zic2 in the retina decreases *Unc5c* expression. Moreover, RGCs that express Zic2 do not project into the contralateral optic nerve [147]. Other guidance mechanisms may act in concert with Unc5c to regulate the projection of RGCs to the opposite eye. In mouse *Slit* and *dicer* mutants or mice lacking specific heparan sulphate synthetic enzymes, the proportion of RGC axons projecting to the opposite eye is increased substantially [84,96,148,149]. Further work, however, will be required to investigate the relative importance and interactions of these disparate signalling mechanisms in the development of direct retina-retina connections.

In silico modelling supports the idea that retina-retina projections are important for the bilateral co-ordination of retinal waves in species where refinement depends upon spontaneous activity. When the size of the target tissue is larger than that of the retina the models indicate that bilateral symmetry of retinotopic mapping depends on synchronisation of activity in both maps (Figure 3C). It remains to be established if retina-retina projections are essential for synchronisation of subsets of waves in both eyes and symmetrical mapping of RGC arbors in both hemispheres of the superior colliculus. However, a comparison between species of *Unc5c* expression, the presence of retina-retina projections and the extent of axonal refinement in visual targets supports this idea. In zebrafish, in which RGC axons project directly to their final location in the tectum, independent of activity, a retina-retina projection is not evident and *Unc5c* is not detected in the retina. In contrast, mice, chicken and ferrets, in which activity-dependent axonal refinement is crucial for establishing RGC axon termination zones, all have axons that project to the opposite retina, and the size of this projection correlates with the extent of *Unc5c* expression in the retina [147]. Taken as a whole these findings suggest a direct connection between both eyes modulates the synchronisation of topographic mapping on both sides of the superior colliculus, and identify Unc5c as a key regulator of the establishment of this connection. Further work, however, will be required to support this idea.

## 9. Conclusions

Considerable progress has been made in understanding the mechanisms that specify ipsilaterally versus contralaterally projecting RGCs, and that guide growth of their axons to targets on both sides of the brain. However, it is clear that much remains to be discovered. For example, recent microarray gene profiling of ipsilaterally versus contralaterally projecting RGCs identified over 300 genes that are differentially expressed between these two cell populations [13], the function of many of which is not known. A key challenge for the future will be establishing the importance of these genes for establishment of the binocular visual pathways. Recent work also has identified novel guidance mechanisms at the chiasm that direct some axons away from the optic tracts into the contralateral optic nerve, and a potential function for this retina-retina projection in co-ordination of axonal refinement in visual targets in both brain hemispheres. Clearly we still have more to learn about the mechanisms regulating the establishment of functional visual pathways. Fully understanding how RGC axon projections are established during development will be important for understanding the basis of conditions such as albinism in which visual projections develop abnormally, and for formulation of new strategies for repair or “re-wiring” of visual projections following disease or damage to the adult visual system.

## References

[1] Masland R.H. (2012). The neuronal organization of the retina. Neuron.

[2] Sanes J.R., Masland R.H. (2015). The types of retinal ganglion cells: Current status and implications for neuronal classification. Annu. Rev. Neurosci..

[3] Baden T., Berens P., Franke K., Roman Roson M., Bethge M., Euler T. (2016). The functional diversity of retinal ganglion cells in the mouse. Nature.

[4] Rheaume B.A., Jereen A., Bolisetty M., Sajid M.S., Yang Y., Renna K., Sun L., Robson P., Trakhtenberg E.F. (2018). Author Correction: Single cell transcriptome profiling of retinal ganglion cells identifies cellular subtypes. Nat. Commun..

[5] Jeffery G., Erskine L. (2005). Variations in the architecture and development of the vertebrate optic chiasm. Prog. Retin. Eye Res..

[6] Morin L.P., Studholme K.M. (2014). Retinofugal projections in the mouse. J. Comp. Neurol..

[7] Feldheim D.A., O’Leary D.D. (2010). Visual map development: Bidirectional signaling, bifunctional guidance molecules, and competition. Cold Spring Harb. Perspect. Biol..

[8] Erskine L., Reijntjes S., Pratt T., Denti L., Schwarz Q., Vieira J.M., Alakakone B., Shewan D., Ruhrberg C. (2011). VEGF signaling through neuropilin 1 guides commissural axon crossing at the optic chiasm. Neuron.

[9] Herrera E., Brown L., Aruga J., Rachel R.A., Dolen G., Mikoshiba K., Brown S., Mason C.A. (2003). Zic2 patterns binocular vision by specifying the uncrossed retinal projection. Cell.

[10] Kuwajima T., Yoshida Y., Takegahara N., Petros T.J., Kumanogoh A., Jessell T.M., Sakurai T., Mason C. (2012). Optic chiasm presentation of Semaphorin6D in the context of Plexin-A1 and Nr-CAM promotes retinal axon midline crossing. Neuron.

[11] Williams S.E., Grumet M., Colman D.R., Henkemeyer M., Mason C.A., Sakurai T. (2006). A role for Nr-CAM in the patterning of binocular visual pathways. Neuron.

[12] Williams S.E., Mann F., Erskine L., Sakurai T., Wei S., Rossi D.J., Gale N.W., Holt C.E., Mason C.A., Henkemeyer M. (2003). Ephrin-B2 and EphB1 mediate retinal axon divergence at the optic chiasm. Neuron.

[13] Wang Q., Marcucci F., Cerullo I., Mason C. (2016). Ipsilateral and Contralateral Retinal Ganglion Cells Express Distinct Genes during Decussation at the Optic Chiasm. eNeuro.

[14] Holt C.E., Bertsch T.W., Ellis H.M., Harris W.A. (1988). Cellular determination in the Xenopus retina is independent of lineage and birth date. Neuron.

[15] Turner D.L., Snyder E.Y., Cepko C.L. (1990). Lineage-independent determination of cell type in the embryonic mouse retina. Neuron.

[16] Wetts R., Fraser S.E. (1988). Multipotent precursors can give rise to all major cell types of the frog retina. Science.

[17] Young R.W. (1985). Cell differentiation in the retina of the mouse. Anat. Rec..

[18] Pacal M., Bremner R. (2014). Induction of the ganglion cell differentiation program in human retinal progenitors before cell cycle exit. Dev. Dyn..

[19] Drager U.C. (1985). Birth dates of retinal ganglion cells giving rise to the crossed and uncrossed optic projections in the mouse. Proc. R. Soc. Lond. B Biol. Sci..

[20] Hufnagel R.B., Le T.T., Riesenberg A.L., Brown N.L. (2010). Neurog2 controls the leading edge of neurogenesis in the mammalian retina. Dev. Biol..

[21] Brzezinski J.A.t., Prasov L., Glaser T. (2012). Math5 defines the ganglion cell competence state in a subpopulation of retinal progenitor cells exiting the cell cycle. Dev. Biol..

[22] Buenaventura D.F., Ghinia-Tegla M.G., Emerson M.M. (2018). Fate-restricted retinal progenitor cells adopt a molecular profile and spatial position distinct from multipotent progenitor cells. Dev. Biol..

[23] De la Huerta I., Kim I.J., Voinescu P.E., Sanes J.R. (2012). Direction-selective retinal ganglion cells arise from molecularly specified multipotential progenitors. Proc. Natl. Acad. Sci. USA.

[24] Hafler B.P., Surzenko N., Beier K.T., Punzo C., Trimarchi J.M., Kong J.H., Cepko C.L. (2012). Transcription factor Olig2 defines subpopulations of retinal progenitor cells biased toward specific cell fates. Proc. Natl. Acad. Sci. USA.

[25] Rompani S.B., Cepko C.L. (2008). Retinal progenitor cells can produce restricted subsets of horizontal cells. Proc. Natl. Acad. Sci. USA.

[26] Davis N., Mor E., Ashery-Padan R. (2011). Roles for Dicer1 in the patterning and differentiation of the optic cup neuroepithelium. Development.

[27] Georgi S.A., Reh T.A. (2010). Dicer is required for the transition from early to late progenitor state in the developing mouse retina. J. Neurosci..

[28] La Torre A., Georgi S., Reh T.A. (2013). Conserved microRNA pathway regulates developmental timing of retinal neurogenesis. Proc. Natl. Acad. Sci. USA.

[29] Dorsky R.I., Rapaport D.H., Harris W.A. (1995). Xotch inhibits cell differentiation in the Xenopus retina. Neuron.

[30] Henrique D., Hirsinger E., Adam J., Le Roux I., Pourquie O., Ish-Horowicz D., Lewis J. (1997). Maintenance of neuroepithelial progenitor cells by Delta-Notch signalling in the embryonic chick retina. Curr. Biol..

[31] Austin C.P., Feldman D.E., Ida J.A., Cepko C.L. (1995). Vertebrate retinal ganglion cells are selected from competent progenitors by the action of Notch. Development.

[32] Esteve P., Sandonis A., Cardozo M., Malapeira J., Ibanez C., Crespo I., Marcos S., Gonzalez-Garcia S., Toribio M.L., Arribas J. (2011). SFRPs act as negative modulators of ADAM10 to regulate retinal neurogenesis. Nat. Neurosci..

[33] Sawant O.B., Jidigam V.K., Fuller R.D., Zucaro O.F., Kpegba C., Yu M., Peachey N.S., Rao S. (2019). The circadian clock gene Bmal1 is required to control the timing of retinal neurogenesis and lamination of Muller glia in the mouse retina. FASEB J..

[34] Gonzalez-Hoyuela M., Barbas J.A., Rodriguez-Tebar A. (2001). The autoregulation of retinal ganglion cell number. Development.

[35] Waid D.K., McLoon S.C. (1998). Ganglion cells influence the fate of dividing retinal cells in culture. Development.

[36] Reh T.A., Hindges R. (2018). MicroRNAs in Retinal Development. Annu. Rev. Vis. Sci..

[37] Martinez-Morales J.R., Del Bene F., Nica G., Hammerschmidt M., Bovolenta P., Wittbrodt J. (2005). Differentiation of the vertebrate retina is coordinated by an FGF signaling center. Dev. Cell.

[38] Masai I., Stemple D.L., Okamoto H., Wilson S.W. (2000). Midline signals regulate retinal neurogenesis in zebrafish. Neuron.

[39] Prada C., Medina J.I., Lopez R., Genis-Galvez J.M., Prada F.A. (1992). Development of retinal displaced ganglion cells in the chick: neurogenesis and morphogenesis. J. Neurosci..

[40] McLoon S.C., Barnes R.B. (1989). Early differentiation of retinal ganglion cells: an axonal protein expressed by premigratory and migrating retinal ganglion cells. J. Neurosci..

[41] Prasov L., Nagy M., Rudolph D.D., Glaser T. (2012). Math5 (Atoh7) gene dosage limits retinal ganglion cell genesis. Neuroreport.

[42] Yang Z., Ding K., Pan L., Deng M., Gan L. (2003). Math5 determines the competence state of retinal ganglion cell progenitors. Dev. Biol..

[43] Brown N.L., Patel S., Brzezinski J., Glaser T. (2001). Math5 is required for retinal ganglion cell and optic nerve formation. Development.

[44] Wang S.W., Kim B.S., Ding K., Wang H., Sun D., Johnson R.L., Klein W.H., Gan L. (2001). Requirement for math5 in the development of retinal ganglion cells. Genes Dev..

[45] Gao Z., Mao C.A., Pan P., Mu X., Klein W.H. (2014). Transcriptome of Atoh7 retinal progenitor cells identifies new Atoh7-dependent regulatory genes for retinal ganglion cell formation. Dev. Neurobiol..

[46] Erkman L., Yates P.A., McLaughlin T., McEvilly R.J., Whisenhunt T., O’Connell S.M., Krones A.I., Kirby M.A., Rapaport D.H., Bermingham J.R. (2000). A POU domain transcription factor-dependent program regulates axon pathfinding in the vertebrate visual system. Neuron.

[47] Pan L., Deng M., Xie X., Gan L. (2008). ISL1 and BRN3B co-regulate the differentiation of murine retinal ganglion cells. Development.

[48] Wu F., Kaczynski T.J., Sethuramanujam S., Li R., Jain V., Slaughter M., Mu X. (2015). Two transcription factors, Pou4f2 and Isl1, are sufficient to specify the retinal ganglion cell fate. Proc. Natl. Acad. Sci. USA.

[49] Chang K.C., Hertz J., Zhang X., Jin X.L., Shaw P., Derosa B.A., Li J.Y., Venugopalan P., Valenzuela D.A., Patel R.D. (2017). Novel Regulatory Mechanisms for the SoxC Transcriptional Network Required for Visual Pathway Development. J. Neurosci..

[50] Johns P.R. (1977). Growth of the adult goldfish eye. III. Source of the new retinal cells. J. Comp. Neurol..

[51] Straznicky K., Gaze R.M. (1971). The growth of the retina in Xenopus laevis: an autoradiographic study. J. Embryol. Exp. Morphol..

[52] Wetts R., Serbedzija G.N., Fraser S.E. (1989). Cell lineage analysis reveals multipotent precursors in the ciliary margin of the frog retina. Dev. Biol..

[53] Belanger M.C., Robert B., Cayouette M. (2017). Msx1-Positive Progenitors in the Retinal Ciliary Margin Give Rise to Both Neural and Non-neural Progenies in Mammals. Dev. Cell.

[54] Marcucci F., Murcia-Belmonte V., Wang Q., Coca Y., Ferreiro-Galve S., Kuwajima T., Khalid S., Ross M.E., Mason C., Herrera E. (2016). The Ciliary Margin Zone of the Mammalian Retina Generates Retinal Ganglion Cells. Cell Rep..

[55] Kiyama T., Long Y., Chen C.K., Whitaker C.M., Shay A., Wu H., Badea T.C., Mohsenin A., Parker-Thornburg J., Klein W.H. (2019). Essential Roles of Tbr1 in the Formation and Maintenance of the Orientation-Selective J-RGCs and a Group of OFF-Sustained RGCs in Mouse. Cell Rep..

[56] Badea T.C., Cahill H., Ecker J., Hattar S., Nathans J. (2009). Distinct roles of transcription factors brn3a and brn3b in controlling the development, morphology, and function of retinal ganglion cells. Neuron.

[57] Badea T.C., Nathans J. (2011). Morphologies of mouse retinal ganglion cells expressing transcription factors Brn3a, Brn3b, and Brn3c: analysis of wild type and mutant cells using genetically-directed sparse labeling. Vision Res..

[58] Sajgo S., Ghinia M.G., Brooks M., Kretschmer F., Chuang K., Hiriyanna S., Wu Z., Popescu O., Badea T.C. (2017). Molecular codes for cell type specification in Brn3 retinal ganglion cells. Proc. Natl. Acad. Sci. USA.

[59] Shi M., Kumar S.R., Motajo O., Kretschmer F., Mu X., Badea T.C. (2013). Genetic interactions between Brn3 transcription factors in retinal ganglion cell type specification. PLoS ONE.

[60] Muzyka V.V., Brooks M., Badea T.C. (2018). Postnatal developmental dynamics of cell type specification genes in Brn3a/Pou4f1 Retinal Ganglion Cells. Neural Dev..

[61] Hong Y.K., Kim I.J., Sanes J.R. (2011). Stereotyped axonal arbors of retinal ganglion cell subsets in the mouse superior colliculus. J. Comp. Neurol..

[62] Colello R.J., Guillery R.W. (1990). The early development of retinal ganglion cells with uncrossed axons in the mouse: retinal position and axonal course. Development.

[63] Guillery R.W., Mason C.A., Taylor J.S. (1995). Developmental determinants at the mammalian optic chiasm. J. Neurosci..

[64] Marcus R.C., Mason C.A. (1995). The first retinal axon growth in the mouse optic chiasm: axon patterning and the cellular environment. J. Neurosci..

[65] Soares C.A., Mason C.A. (2015). Transient ipsilateral retinal ganglion cell projections to the brain: Extent, targeting, and disappearance. Dev. Neurobiol..

[66] Pak W., Hindges R., Lim Y.S., Pfaff S.L., O’Leary D.D. (2004). Magnitude of binocular vision controlled by islet-2 repression of a genetic program that specifies laterality of retinal axon pathfinding. Cell.

[67] Marcucci F., Soares C.A., Mason C. (2019). Distinct timing of neurogenesis of ipsilateral and contralateral retinal ganglion cells. J. Comp. Neurol..

[68] Bhansali P., Rayport I., Rebsam A., Mason C. (2014). Delayed neurogenesis leads to altered specification of ventrotemporal retinal ganglion cells in albino mice. Neural. Dev..

[69] Ilia M., Jeffery G. (1996). Delayed neurogenesis in the albino retina: evidence of a role for melanin in regulating the pace of cell generation. Brain Res. Dev. Brain Res..

[70] Rachel R.A., Dolen G., Hayes N.L., Lu A., Erskine L., Nowakowski R.S., Mason C.A. (2002). Spatiotemporal features of early neuronogenesis differ in wild-type and albino mouse retina. J. Neurosci..

[71] Webster M.J., Rowe M.H. (1991). Disruption of developmental timing in the albino rat retina. J. Comp. Neurol..

[72] Garcia-Frigola C., Carreres M.I., Vegar C., Mason C., Herrera E. (2008). Zic2 promotes axonal divergence at the optic chiasm midline by EphB1-dependent and -independent mechanisms. Development.

[73] Carreres M.I., Escalante A., Murillo B., Chauvin G., Gaspar P., Vegar C., Herrera E. (2011). Transcription factor Foxd1 is required for the specification of the temporal retina in mammals. J. Neurosci..

[74] Herrera E., Marcus R., Li S., Williams S.E., Erskine L., Lai E., Mason C. (2004). Foxd1 is required for proper formation of the optic chiasm. Development.

[75] Cho S.H., Cepko C.L. (2006). Wnt2b/beta-catenin-mediated canonical Wnt signaling determines the peripheral fates of the chick eye. Development.

[76] Iwai-Takekoshi L., Balasubramanian R., Sitko A., Khan R., Weinreb S., Robinson K., Mason C. (2018). Activation of Wnt signaling reduces ipsilaterally projecting retinal ganglion cells in pigmented retina. Development.

[77] Sanchez-Arrones L., Nieto-Lopez F., Sanchez-Camacho C., Carreres M.I., Herrera E., Okada A., Bovolenta P. (2013). Shh/Boc signaling is required for sustained generation of ipsilateral projecting ganglion cells in the mouse retina. J. Neurosci..

[78] Fabre P.J., Shimogori T., Charron F. (2010). Segregation of ipsilateral retinal ganglion cell axons at the optic chiasm requires the Shh receptor Boc. J. Neurosci..

[79] Young T.R., Bourke M., Zhou X., Oohashi T., Sawatari A., Fassler R., Leamey C.A. (2013). Ten-m2 is required for the generation of binocular visual circuits. J. Neurosci..

[80] Triplett J.W., Wei W., Gonzalez C., Sweeney N.T., Huberman A.D., Feller M.B., Feldheim D.A. (2014). Dendritic and axonal targeting patterns of a genetically-specified class of retinal ganglion cells that participate in image-forming circuits. Neural Dev..

[81] Kuwajima T., Soares C.A., Sitko A.A., Lefebvre V., Mason C. (2017). SoxC Transcription Factors Promote Contralateral Retinal Ganglion Cell Differentiation and Axon Guidance in the Mouse Visual System. Neuron.

[82] Quina L.A., Pak W., Lanier J., Banwait P., Gratwick K., Liu Y., Velasquez T., O’Leary D.D., Goulding M., Turner E.E. (2005). Brn3a-expressing retinal ganglion cells project specifically to thalamocortical and collicular visual pathways. J. Neurosci..

[83] Erskine L., Williams S.E., Brose K., Kidd T., Rachel R.A., Goodman C.S., Tessier-Lavigne M., Mason C.A. (2000). Retinal ganglion cell axon guidance in the mouse optic chiasm: expression and function of robos and slits. J. Neurosci..

[84] Plump A.S., Erskine L., Sabatier C., Brose K., Epstein C.J., Goodman C.S., Mason C.A., Tessier-Lavigne M. (2002). Slit1 and Slit2 cooperate to prevent premature midline crossing of retinal axons in the mouse visual system. Neuron.

[85] Nakagawa S., Brennan C., Johnson K.G., Shewan D., Harris W.A., Holt C.E. (2000). Ephrin-B regulates the Ipsilateral routing of retinal axons at the optic chiasm. Neuron.

[86] Peng J., Fabre P.J., Dolique T., Swikert S.M., Kermasson L., Shimogori T., Charron F. (2018). Sonic Hedgehog Is a Remotely Produced Cue that Controls Axon Guidance Trans-axonally at a Midline Choice Point. Neuron.

[87] Chan S.O., Chung K.Y., Taylor J.S. (1999). The effects of early prenatal monocular enucleation on the routing of uncrossed retinofugal axons and the cellular environment at the chiasm of mouse embryos. Eur. J. Neurosci..

[88] Godement P., Salaun J., Metin C. (1987). Fate of uncrossed retinal projections following early or late prenatal monocular enucleation in the mouse. J. Comp. Neurol..

[89] Guillery R.W. (1989). Early monocular enucleations in fetal ferrets produce a decrease of uncrossed and an increase of crossed retinofugal components: a possible model for the albino abnormality. J. Anat..

[90] Lustig M., Erskine L., Mason C.A., Grumet M., Sakurai T. (2001). Nr-CAM expression in the developing mouse nervous system: ventral midline structures, specific fiber tracts, and neuropilar regions. J. Comp. Neurol..

[91] Ruhrberg C., Gerhardt H., Golding M., Watson R., Ioannidou S., Fujisawa H., Betsholtz C., Shima D.T. (2002). Spatially restricted patterning cues provided by heparin-binding VEGF-A control blood vessel branching morphogenesis. Genes Dev..

[92] Tillo M., Erskine L., Cariboni A., Fantin A., Joyce A., Denti L., Ruhrberg C. (2015). VEGF189 binds NRP1 and is sufficient for VEGF/NRP1-dependent neuronal patterning in the developing brain. Development.

[93] Erskine L., Francois U., Denti L., Joyce A., Tillo M., Bruce F., Vargesson N., Ruhrberg C. (2017). VEGF-A and neuropilin 1 (NRP1) shape axon projections in the developing CNS via dual roles in neurons and blood vessels. Development.

[94] Sitko A.A., Kuwajima T., Mason C.A. (2018). Eye-specific segregation and differential fasciculation of developing retinal ganglion cell axons in the mouse visual pathway. J. Comp. Neurol..

[95] Colello S.J., Coleman L.A. (1997). Changing course of growing axons in the optic chiasm of the mouse. J. Comp. Neurol..

[96] Pinter R., Hindges R. (2010). Perturbations of microRNA function in mouse dicer mutants produce retinal defects and lead to aberrant axon pathfinding at the optic chiasm. PLoS ONE.

[97] Dell A.L., Fried-Cassorla E., Xu H., Raper J.A. (2013). cAMP-induced expression of neuropilin1 promotes retinal axon crossing in the zebrafish optic chiasm. J. Neurosci..

[98] Sakai J.A., Halloran M.C. (2006). Semaphorin 3d guides laterality of retinal ganglion cell projections in zebrafish. Development.

[99] Panza P., Sitko A.A., Maischein H.M., Koch I., Flotenmeyer M., Wright G.J., Mandai K., Mason C.A., Sollner C. (2015). The LRR receptor Islr2 is required for retinal axon routing at the vertebrate optic chiasm. Neural Dev..

[100] Sparks D.L., Lee C., Rohrer W.H. (1990). Population coding of the direction, amplitude, and velocity of saccadic eye movements by neurons in the superior colliculus. Cold Spring Harb. Symp. Quant. Biol..

[101] Liang F., Xiong X.R., Zingg B., Ji X.Y., Zhang L.I., Tao H.W. (2015). Sensory Cortical Control of a Visually Induced Arrest Behavior via Corticotectal Projections. Neuron.

[102] Shang C., Liu Z., Chen Z., Shi Y., Wang Q., Liu S., Li D., Cao P. (2015). BRAIN CIRCUITS. A parvalbumin-positive excitatory visual pathway to trigger fear responses in mice. Science.

[103] Wei P., Liu N., Zhang Z., Liu X., Tang Y., He X., Wu B., Zhou Z., Liu Y., Li J. (2015). Corrigendum: Processing of visually evoked innate fear by a non-canonical thalamic pathway. Nat. Commun..

[104] Plas D.T., Lopez J.E., Crair M.C. (2005). Pretarget sorting of retinocollicular axons in the mouse. J. Comp. Neurol..

[105] Cioni J.M., Wong H.H., Bressan D., Kodama L., Harris W.A., Holt C.E. (2018). Axon-Axon Interactions Regulate Topographic Optic Tract Sorting via CYFIP2-Dependent WAVE Complex Function. Neuron.

[106] Osterhout J.A., El-Danaf R.N., Nguyen P.L., Huberman A.D. (2014). Birthdate and outgrowth timing predict cellular mechanisms of axon target matching in the developing visual pathway. Cell Rep..

[107] Cang J., Feldheim D.A. (2013). Developmental mechanisms of topographic map formation and alignment. Annu. Rev. Neurosci..

[108] Thompson A., Gribizis A., Chen C., Crair M.C. (2017). Activity-dependent development of visual receptive fields. Curr. Opin. Neurobiol..

[109] Ellis E.M., Gauvain G., Sivyer B., Murphy G.J. (2016). Shared and distinct retinal input to the mouse superior colliculus and dorsal lateral geniculate nucleus. J. Neurophysiol..

[110] Basso M.A., May P.J. (2017). Circuits for Action and Cognition: A View from the Superior Colliculus. Annu. Rev. Vis. Sci..

[111] May P.J. (2006). The mammalian superior colliculus: laminar structure and connections. Prog. Brain Res..

[112] Drager U.C., Olsen J.F. (1980). Origins of crossed and uncrossed retinal projections in pigmented and albino mice. J. Comp. Neurol..

[113] Wang Q., Burkhalter A. (2013). Stream-related preferences of inputs to the superior colliculus from areas of dorsal and ventral streams of mouse visual cortex. J. Neurosci..

[114] Huberman A.D., Feller M.B., Chapman B. (2008). Mechanisms underlying development of visual maps and receptive fields. Annu. Rev. Neurosci..

[115] Cang J., Kaneko M., Yamada J., Woods G., Stryker M.P., Feldheim D.A. (2005). Ephrin-as guide the formation of functional maps in the visual cortex. Neuron.

[116] Rashid T., Upton A.L., Blentic A., Ciossek T., Knoll B., Thompson I.D., Drescher U. (2005). Opposing gradients of ephrin-As and EphA7 in the superior colliculus are essential for topographic mapping in the mammalian visual system. Neuron.

[117] Triplett J.W., Phan A., Yamada J., Feldheim D.A. (2012). Alignment of multimodal sensory input in the superior colliculus through a gradient-matching mechanism. J. Neurosci..

[118] Drescher U., Kremoser C., Handwerker C., Loschinger J., Noda M., Bonhoeffer F. (1995). In vitro guidance of retinal ganglion cell axons by RAGS, a 25 kDa tectal protein related to ligands for Eph receptor tyrosine kinases. Cell.

[119] Monschau B., Kremoser C., Ohta K., Tanaka H., Kaneko T., Yamada T., Handwerker C., Hornberger M.R., Loschinger J., Pasquale E.B. (1997). Shared and distinct functions of RAGS and ELF-1 in guiding retinal axons. EMBO J..

[120] Nakamoto M., Cheng H.J., Friedman G.C., McLaughlin T., Hansen M.J., Yoon C.H., O’Leary D.D., Flanagan J.G. (1996). Topographically specific effects of ELF-1 on retinal axon guidance in vitro and retinal axon mapping in vivo. Cell.

[121] Brown A., Yates P.A., Burrola P., Ortuno D., Vaidya A., Jessell T.M., Pfaff S.L., O’Leary D.D., Lemke G. (2000). Topographic mapping from the retina to the midbrain is controlled by relative but not absolute levels of EphA receptor signaling. Cell.

[122] Cang J., Wang L., Stryker M.P., Feldheim D.A. (2008). Roles of ephrin-as and structured activity in the development of functional maps in the superior colliculus. J. Neurosci..

[123] Feldheim D.A., Kim Y.I., Bergemann A.D., Frisen J., Barbacid M., Flanagan J.G. (2000). Genetic analysis of ephrin-A2 and ephrin-A5 shows their requirement in multiple aspects of retinocollicular mapping. Neuron.

[124] Frisen J., Yates P.A., McLaughlin T., Friedman G.C., O’Leary D.D., Barbacid M. (1998). Ephrin-A5 (AL-1/RAGS) is essential for proper retinal axon guidance and topographic mapping in the mammalian visual system. Neuron.

[125] Pfeiffenberger C., Yamada J., Feldheim D.A. (2006). Ephrin-As and patterned retinal activity act together in the development of topographic maps in the primary visual system. J. Neurosci..

[126] Triplett J.W., Owens M.T., Yamada J., Lemke G., Cang J., Stryker M.P., Feldheim D.A. (2009). Retinal input instructs alignment of visual topographic maps. Cell.

[127] Hindges R., McLaughlin T., Genoud N., Henkemeyer M., O’Leary D. (2002). EphB forward signaling controls directional branch extension and arborization required for dorsal-ventral retinotopic mapping. Neuron.

[128] Thakar S., Chenaux G., Henkemeyer M. (2011). Critical roles for EphB and ephrin-B bidirectional signalling in retinocollicular mapping. Nat. Commun..

[129] McLaughlin T., Torborg C.L., Feller M.B., O’Leary D.D. (2003). Retinotopic map refinement requires spontaneous retinal waves during a brief critical period of development. Neuron.

[130] Assali A., Gaspar P., Rebsam A. (2014). Activity dependent mechanisms of visual map formation—from retinal waves to molecular regulators. Semin. Cell Dev. Biol..

[131] Godement P., Salaun J., Imbert M. (1984). Prenatal and postnatal development of retinogeniculate and retinocollicular projections in the mouse. J. Comp. Neurol..

[132] Jaubert-Miazza L., Green E., Lo F.S., Bui K., Mills J., Guido W. (2005). Structural and functional composition of the developing retinogeniculate pathway in the mouse. Vis. Neurosci..

[133] Dhande O.S., Hua E.W., Guh E., Yeh J., Bhatt S., Zhang Y., Ruthazer E.S., Feller M.B., Crair M.C. (2011). Development of single retinofugal axon arbors in normal and beta2 knock-out mice. J. Neurosci..

[134] Ackman J.B., Burbridge T.J., Crair M.C. (2012). Retinal waves coordinate patterned activity throughout the developing visual system. Nature.

[135] Feller M.B., Wellis D.P., Stellwagen D., Werblin F.S., Shatz C.J. (1996). Requirement for cholinergic synaptic transmission in the propagation of spontaneous retinal waves. Science.

[136] Benjumeda I., Escalante A., Law C., Morales D., Chauvin G., Muca G., Coca Y., Marquez J., Lopez-Bendito G., Kania A. (2013). Uncoupling of EphA/ephrinA signaling and spontaneous activity in neural circuit wiring. J. Neurosci..

[137] Chandrasekaran A.R., Plas D.T., Gonzalez E., Crair M.C. (2005). Evidence for an instructive role of retinal activity in retinotopic map refinement in the superior colliculus of the mouse. J. Neurosci..

[138] Rebsam A., Petros T.J., Mason C.A. (2009). Switching retinogeniculate axon laterality leads to normal targeting but abnormal eye-specific segregation that is activity dependent. J. Neurosci..

[139] Rossi F.M., Pizzorusso T., Porciatti V., Marubio L.M., Maffei L., Changeux J.P. (2001). Requirement of the nicotinic acetylcholine receptor beta 2 subunit for the anatomical and functional development of the visual system. Proc. Natl. Acad. Sci. USA.

[140] Garcia-Frigola C., Herrera E. (2010). Zic2 regulates the expression of Sert to modulate eye-specific refinement at the visual targets. EMBO J..

[141] Failor S.W., Ng A., Cheng H.J. (2018). Monocular enucleation alters retinal waves in the surviving eye. Neural Dev..

[142] Avellaneda-Chevrier V.K., Wang X., Hooper M.L., Chauhan B.C. (2015). The retino-retinal projection: Tracing retinal ganglion cells projecting to the contralateral retina. Neurosci. Lett..

[143] Braekevelt C.R., Beazley L.D., Dunlop S.A., Darby J.E. (1986). Numbers of axons in the optic nerve and of retinal ganglion cells during development in the marsupial Setonix brachyurus. Brain Res..

[144] Bunt S.M., Lund R.D. (1981). Development of a transient retino-retinal pathway in hooded and albino rats. Brain Res..

[145] McLoon S.C., Lund R.D. (1982). Transient retinofugal pathways in the developing chick. Exp. Brain Res..

[146] Nadal-Nicolas F.M., Valiente-Soriano F.J., Salinas-Navarro M., Jimenez-Lopez M., Vidal-Sanz M., Agudo-Barriuso M. (2015). Retino-retinal projection in juvenile and young adult rats and mice. Exp. Eye Res..

[147] Murcia-Belmonte V., Coca Y., Vegar C., Negueruela S., de Juan Romero C., Valino A.J., Sala S., DaSilva R., Kania A., Borrell V. (2019). A Retino-retinal Projection Guided by Unc5c Emerged in Species with Retinal Waves. Curr. Biol..

[148] Inatani M., Irie F., Plump A.S., Tessier-Lavigne M., Yamaguchi Y. (2003). Mammalian brain morphogenesis and midline axon guidance require heparan sulfate. Science.

[149] Pratt T., Conway C.D., Tian N.M., Price D.J., Mason J.O. (2006). Heparan sulphation patterns generated by specific heparan sulfotransferase enzymes direct distinct aspects of retinal axon guidance at the optic chiasm. J. Neurosci..

